# Axon numbers and landmarks of trigeminal donor nerves for corneal neurotization

**DOI:** 10.1371/journal.pone.0206642

**Published:** 2018-10-31

**Authors:** Eva Györi, Chieh-Han John Tzou, Wolfgang J. Weninger, Lukas Reissig, Ursula Schmidt-Erfurth, Christine Radtke, Roman Dunavoelgyi

**Affiliations:** 1 Division of Plastic and Reconstructive Surgery, Department of Surgery, Medical University of Vienna, Vienna, Austria; 2 Division of Anatomy, Medical University of Vienna, Vienna, Austria; 3 Department of Ophthalmology and Optometry, Medical University of Vienna, Vienna, Austria; BG Trauma Center Ludwigshafen, GERMANY

## Abstract

**Purpose:**

Corneal anesthesia leads to chronic corneal injury. This anatomical study characterizes the donor nerve branches of the supratrochlear and supraorbital nerves used for corneal neurotization.

**Methods:**

In 13 non-embalmed cadavers, the supratrochlear and supraorbital nerves were dissected and distances to anatomical landmarks measured. Cross-sections of supratrochlear and supraorbital donor nerves were harvested and histomorphometrically analyzed to assess the number of myelinated axons.

**Results:**

The donor axon counts were 3146 ± 1069.9 for the supratrochlear and 1882 ± 903 for the supraorbital nerve distal to the supraorbital notch. The supratrochlear nerve was dissected on the medial upper eyelid 2 cm lateral to the facial midline and the branch of the supraorbital nerve 1 cm medial to the mid-pupillary line.

**Conclusion:**

The supraorbital and supratrochlear branches of the trigeminal nerve are potent donor nerves for corneal neurotization in the treatment of neuropathic keratopathy and can be reliably dissected using anatomical landmarks.

## Introduction

Corneal anesthesia is a serious condition that leads to corneal injury through impaired protective sensation, which causes chronic erosions and infections, which may result in loss of vision in severe cases [1]. The cornea is innervated by the ophthalmic division of the trigeminal nerve [2]. Corneal sensitivity is important for initiating the blink reflex and preserving the integrity of the corneal epithelium [3].

Multiple congenital and acquired etiologies of corneal anesthesia have been identified, which lead to the clinical condition described as neurotrophic keratopathy [1, 3, 4]. The most common causes of neurotrophic keratopathy are viral infections such as herpes simplex or herpes zoster keratoconjunctivitis [4, 5]. Acquired causes of corneal anesthesia include chemical burns, physical injuries and corneal surgery [4]. Intracranial pathologies affecting the trigeminal nerve or ganglion, lesions in the posterior fossa and pathologies affecting the brainstem can also lead to neurotrophic keratopathy [4]. Systemic conditions like diabetes or demyelinating diseases can also affect the corneal sensitivity [1, 4].

Congenital corneal anesthesia is either complete or partial, and typically affects both eyes [2, 6]. Cases of unilateral congenital corneal anesthesia have been reported, which can be associated with anesthesia in other areas of the face supplied by the first and second division of the trigeminal nerve [2]. Congenital cases can be either syndromatic, or non-syndromatic. Syndromatic cases can be associated with ocular, neurological and systemic conditions, like the VACTERL association, Goldenhar syndrome or Moebius syndrome [2].

With multiple etiologies causing corneal anesthesia, the cellular pathophysiological mechanisms are complex and not yet fully investigated [4]. The cornea is densely innervated by sensory, sympathetic and parasympathetic nerves, which each express specific neurotransmitters [4]. Substance P and calcitonin gene-related peptide are highly expressed in sensory corneal nerves, while sympathetic nerves contain neurotransmitters like noradrenaline, serotonin and neuropeptide Y [4]. Altered neurotransmitter levels are a crucial mechanism leading to neurothrophic keratopathy [4]. Experimental studies showed that altered levels of cAMP, cGMP and Substance P affect corneal epithelial cell regeneration in corneal anesthesia [2, 7].

Despite the complex pathophysiology of corneal anesthesia, the treatment of neurotrophic keratopathy was mainly symptomatic and included artificial tears, lubricants, bandage contact lenses, protective glasses, punctual occlusion and tarsorrhaphy to avoid severe consequences of corneal injury [2, 8]. Surgical reinnervation of the anesthetic cornea was first described by Terzis and colleagues in 2009. Six patients with unilateral facial palsy and neurotrophic keratopathy were treated with corneal neurotization surgery using direct nerve transfers [3]. Through a bicoronary incision, the contralateral supraorbital and supratrochlear nerves were used as donor nerves [3]. Corneal sensation was restored, but the method did not gain widespread popularity, due to the bicoronary approach and extensive sensory donor nerve deficit [5].

In 2014, Elbaz and colleagues published a minimally-invasive technique where autologous sural nerve grafts are used for corneal neurotization to reduce the extent of surgical exposure and to reduce donor site morbidity [5]. Depending on the etiology of corneal anesthesia, the contralateral or ipsilateral supratrochlear nerves were used as donor nerves, with a minimally-invasive access through small incision in the tarsal folds [5, 9]. This method includes a side-to-end coaptation of a sural nerve graft to the supratrochlear nerve, thus limiting the donor nerve deficit by preserving the continuity of the donor nerve [5, 9]. The authors reported improved corneal sensation 6 months after neurotization, which is determined by the regeneration distance, given peripheral nerve regeneration of approximately 1 mm per day [9]. Postoperative in vivo confocal microscopy showed successful corneal reinnervation in the stromal and subbasal corneal layers [10]. Some of the treated patients subsequently underwent corneal transplantation after successful restoration of corneal sensation [5, 10].

Ting and colleagues described favorable long-term outcomes after corneal neurotization, which were confirmed by in vivo confocal microscopy and histopathological measures [11]. Benkhatar and colleagues successfully used the great auricular nerve as a nerve graft instead of the sural nerve for minimally-invasive corneal neurotization in a patient with unilateral neurotrophic keratopathy [12]. The nerve graft was coapted end-to-end to the contralateral supratrochlear nerve [12]. A minimally-invasive case of corneal neurotization using the ipsilateral supraorbital nerve was described by Jacinto and colleagues in a patient with corneal anesthesia from a local injury to the long ciliary nerves [13]. In this case report, the direct transfer of the supraorbital nerve was performed while avoiding a bicoronary incision. In this case, the interposition of a nerve graft was not necessary, thus avoiding an additional sensory donor nerve deficit and longer regeneration distances [13].

In all reported cases of corneal reinnervation, donor nerve branches of the supratrochlear or the supraorbital nerve, were used [3, 5]. They are distal branches of the ophthalmic division of the trigeminal nerve and provide sensation to the forehead region, the upper eyelid and the cornea [3, 5].

As recent reports of corneal neurotization demonstrated reproducible, successful reinnervation in the anesthetic cornea, the donor nerves have not yet been further characterized. In this study, we aimed to describe the supratrochlear and supraorbital nerves as donor nerves for corneal neurotization and to analyze the number of myelinated axons available for sensory reinnervation of the anesthetic cornea. The detailed analysis of available donor nerve branches is crucial, because their axonal load determines the reinnervation capacity and the subsequent donor nerve deficit. Terzis and colleagues described 900 myelinated axons as a cut-off value for donor nerve branch selection in cases of facial reanimation surgery [14]. However, these values might not directly translate to corneal reinnervation, as donor branches of the facial nerve a motoric donors and regeneration distances through cross-face nerve grafts are significantly higher. The optimal number of donor axons for corneal reinnervation is not yet established. In this anatomical study, we aimed to provide detailed data of trigeminal donor nerve characteristics. The second aim of this study was to describe reliable anatomical landmarks to guide intraoperative donor nerve dissection.

## Materials and methods

The study was performed under the approval of the ethics commission of the Medical University of Vienna (protocol number 1826/2016) and followed the guidelines of the Helsinki Declaration of 1975 and the regulations concerning the use of body donor materials for science and teaching. Written pre-mortem consent for anatomical research was obtained from each individual.

### Anatomical dissection

**Fig 1:**
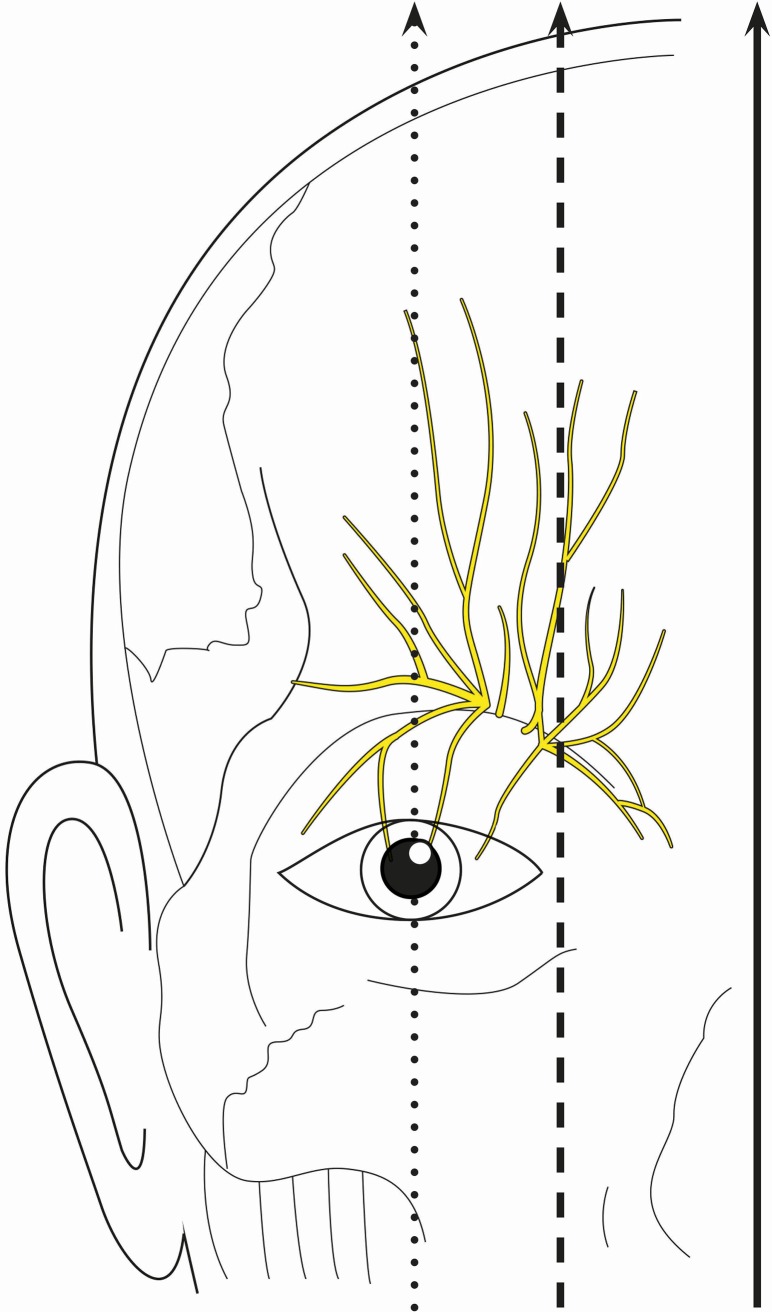
Anatomical landmarks. The facial midline (solid arrow), the medial canthus (dashed arrow) and the mid-pupillary line (dotted arrow) were used as landmarks to provide guiding distances for donor nerve branch dissection of the supraorbital and supratrochlear nerves in this anatomical study.

Thirteen fresh, non-embalmed white cadavers (7 female and 6 male) were dissected for this study. The exclusion criteria for anatomical specimen selection were periorbital scars and cranial trauma. All dissections were performed under surgical loupe magnification. Measurements were obtained bilaterally using a standard surgical ruler. Through a tarsal incision, the supratrochlear nerve was identified and the distances to the facial midline, the medial corner of the eyelid, the mid-pupillary line and the nasocranial margin of the orbita were measured (Fig 1). Subsequently, the supraorbital nerve was dissected using a supraciliar incision. The distances to the facial midline, the medial corner of the eyelid and the mid-pupillary line were measured (Fig 1).

Sural nerve grafts were harvested with minimally invasive technique through a lateral retromalleolar incision using a nerve stripper. Proximally, 2 to 3 small skin incisions of 15 to 20 mm were performed to aid nerve graft harvesting, depending on the branching pattern of the sural nerve [15]. Nerve grafts of approximately 15 cm length were used for corneal neurotization, as previously described by Bains and colleagues [9].

**Fig 2:**
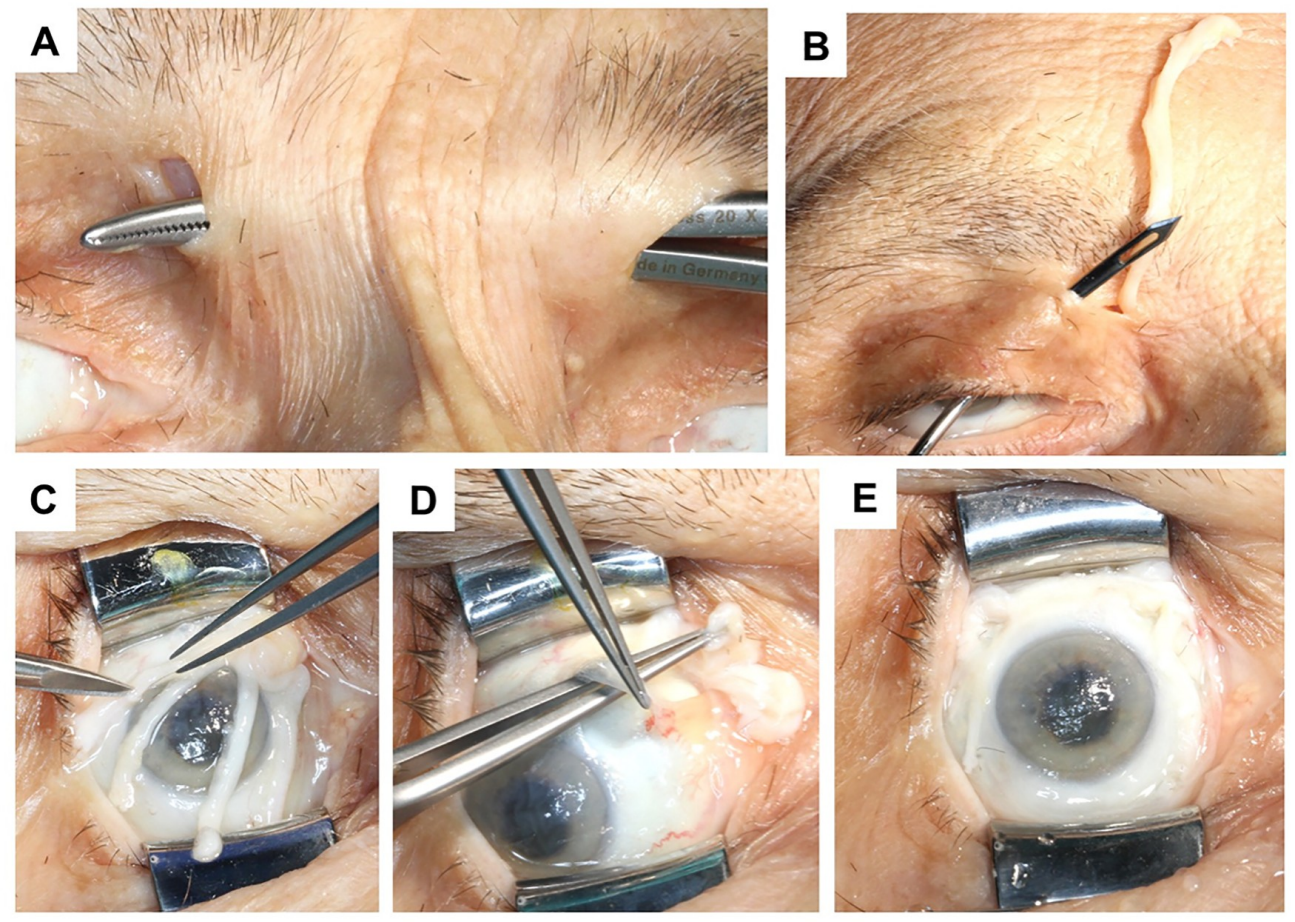
Neurotization of the anesthetic cornea using a sural nerve graft and supratrochlear donor nerve. Cadaveric dissection to neurotize the right cornea using the contralateral supratrochlear nerve as a donor, which is dissected through a small skin incision on the upper eyelid. A subcutaneous tunnel connects the donor and recipient side (A). The sural nerve is used as an autologous nerve graft and pulled through the to the superior fornix with a Wright Fascia Needle (B). The fascicles of the sural nerve graft are separated (C) and tunneled under the conjunctiva (D). The 4 to 5 fascicles are placed around the limbus and sutured to the sclera using 10–0 Nylon interrupted sutures (E).

As clinically described in unilateral cases of neuropathic keratopathy, the contralateral supratrochlear or supraorbital nerve can be used as the donor nerve branches and the sural nerve graft was passed from the donor side to the recipient side through a subcutaneous tunnel (Fig 2A). On the insensate side, the sural nerve graft was pulled through a craniomedial conjunctival incision and guided to the superior fornix using a Wright Fascia Needle (Fig 2B). The fascicles at end of the nerve graft were separated and the ends trimmed (Fig 2C). The 4 to 5 individual fascicles of the nerve graft were placed under the conjunctiva (Fig 2D), spread out evenly and sutured to the sclera with perilimbal 10–0 Nylon interrupted sutures (Fig 2E) as described previously [5, 9]. The nerve graft was coapted to the donor nerve with interrupted 10–0 Nylon epineural sutures. In a clinical situation, the patient’s eye would have been closed with a temporary tarsorrhaphy for one week [5].

### Nerve cross-sections and myelinated donor nerve counts

Cross-sections of the supratrochlear and supraorbital nerves were obtained for histomorphometric analysis. The samples were harvested at the site of coaptation to the autologous nerve graft to assess the available donor nerve counts for corneal reinnervation. Samples of the supraorbital nerve were harvested proximally in the cranial part of the orbita and further distally after its passage through the supraorbital notch. Additionally, biopsies of the sural nerve graft were harvested. Nerve biopsies were fixed in 2.5% glutaraldehyde and processed as previously described [16, 17]. Characteristics of nerve cross-sections and myelinated nerve fibers were measured using a semi-automated image analyzing software (LUCIA-M, Nikon Laboratory Imaging, Prague, Czech Republic) [17]. Axon diameters were marked by the investigator for an area covering at least 30% of the nerve cross-section. The image analysis software was subsequently used to calculate the axon numbers of the entire nerve biopsy.

### Statistical analysis

Mean values and standard deviations were reported for all anatomical measurements and histomorphometric criteria. The different myelinated donor axon counts were analyzed by one-way ANOVA and post-hoc Bonferroni corrections. A two-sided p-value of <0.05 was considered statistically significant.

## Results

### Anatomical dissection

Seven female and 6 male cadavers were dissected for this study. The mean age was 79.4 ± 9.5 years. Cause of death was pneumonia in 3 cases, cardiovascular events in 3 cases and cancer in 1 case. In 6 cases, the exact cause of death was not available, however, all included anatomical specimen met the criteria of no periorbital scars or trauma.

### Anatomical landmarks

**Fig 3:**
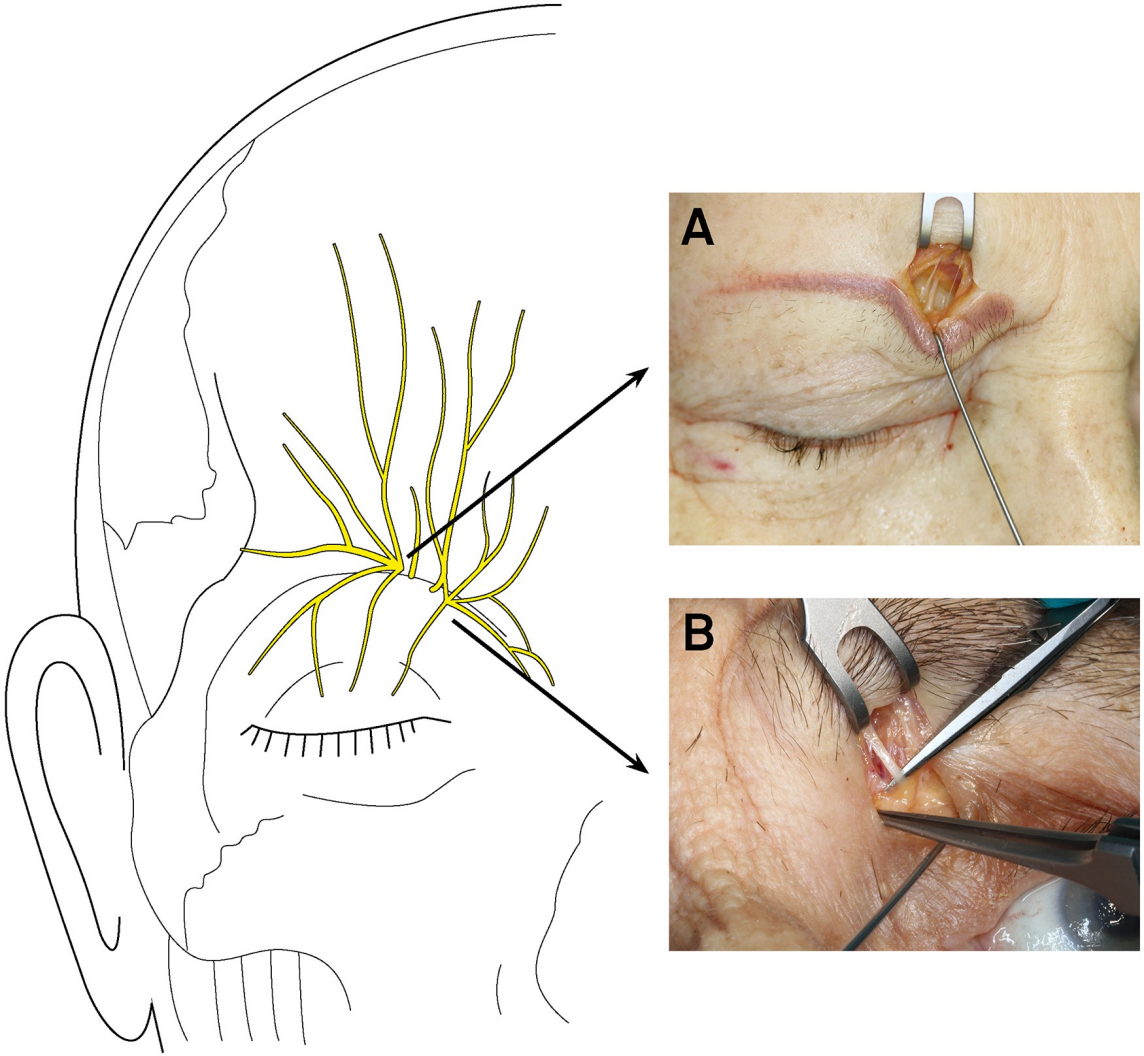
Donor nerve branch dissection using anatomical landmarks. The donor branch of the supraorbital nerve distal to the supraorbital notch accessed through a supraciliary incision and found approximately 1 cm medial to the mid-pupillary line (A). The supratrochlear nerve branch was located 2 cm lateral to the facial midline and dissected through a small transverse incision on the medial upper eyelid (B). The sketch on the left side displays the anatomical landmarks (see Fig 1).

In this cadaver study, the supratrochlear nerve (Fig 3A) was dissected through a short transverse incision on the medial upper eyelid under surgical loupe magnification. The mean distance to the supratrochlear nerve was 20.1 ± 2.8 mm lateral to the facial midline and 14 ± 2.9 mm medial to the mid-pupillary line. The supratrochlear nerve was found 2.7 ± 1.6 mm lateral to the medial canthus and 2 mm caudal to the craniomedial orbital rim.

The supraorbital nerve (Fig 3B) was accessed through a supraciliary incision at the medial third of the eyebrow. The supraorbital nerve was 19.6 ± 3.1 mm lateral to the facial midline and 11.8 ± 2.4 mm medial to the mid-pupillary line. The mean distance to the medial canthus was 1.7 ± 0.6 mm.

As practical dissection guidelines for the supratrochlear and supraorbital nerves as donor nerves with limited skin incisions, the supratrochlear nerve can be located through a tarsal incision approximately 2 cm lateral to the facial midline and 2 mm caudal to the craniomedial orbital rim. The supraorbital nerve can be dissected through a supraciliary incision approximately 1 cm medial to the mid-pupillary line.

### Myelinated donor nerve fiber counts

**Fig 4:**
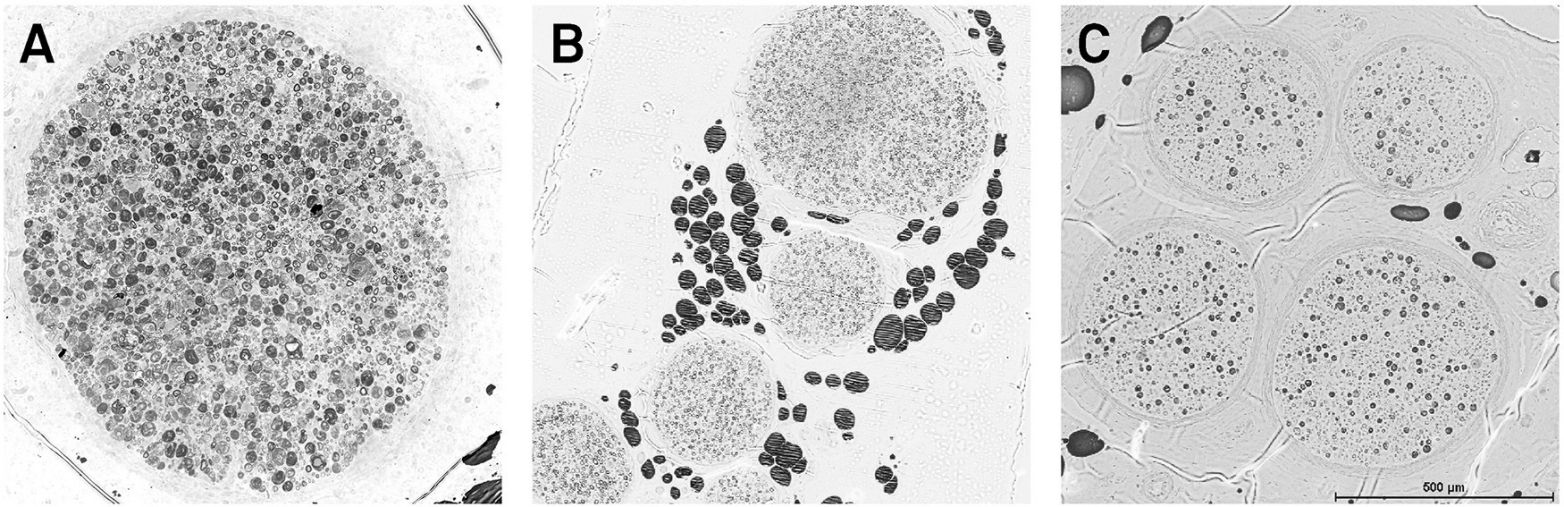
Nerve biopsies. Cross-sections of nerve biopsies of the supratrochlear nerve (A), the supraorbital nerve (B) and the sural nerve graft (C) were analyzed histomorphometrically to determine the myelinated axon counts.

Nerve biopsies were obtained for histomorphometric analysis of myelinated axons and nerve cross-sections were evaluated. The supratrochlear nerve contained 3146 ± 1069.9 myelinated axons in 2 ± 1.5 fascicles (Fig 4A). The supraorbital nerve branch distal to the supraorbital notch, which was dissected through a supraciliary incision, included 1882 ± 903 myelinated axons in 6 ± 3.8 fascicles (Fig 4B). The proximal part of the supraorbital nerve, which was dissected proximal to the orbital rim through a tarsal approach, contained 6035 ± 1675.2 myelinated nerve fibers in 4 ± 2.2 fascicles. The sural nerves harvested as autologous nerve grafts contained 3179 ± 1524.5 myelinated nerve fibers in 7 ± 3.8 fascicles (Fig 4C).

The myelinated axon counts of cross-sections of the proximal supraorbital nerves were significantly higher than those of the distal supraorbital nerves (p < 0.001), the supratrochlear nerves (p = 0.003) and the sural nerves (p = 0.001). The myelinated axon counts of the supratrochlear nerve and the distal supraorbital nerve biopsies did not differ significantly.

## Discussion

Reinnervating the anesthetic cornea provides a therapeutic treatment option for neuropathic keratopathy, which was previously managed symptomatically [3, 5, 9]. Corneal neurotization was first described by Terzis and colleagues and has been successfully performed in few patients worldwide [3, 9, 11]. Corneal reinnervation is either performed by direct nerve transfers [3, 13] or by interposition of nerve grafts [5, 9, 11, 12]. This study analyzed the characteristics of the supratrochlear and supraorbital nerve branches of the trigeminal nerve used as donors for corneal neurotization. Additionally, reliable anatomical landmarks for donor nerve branch dissection were described for both, the supratrochlear and the supraorbital nerve branches.

The main findings of this study were the robust donor nerve counts of the trigeminal donor nerve branches: the supratrochlear nerve contained approximately 3150 myelinated axons and the supraorbital nerve 1880 distal and 6000 proximal to the supraorbital notch, respectively. The high numbers of myelinated axons available for sensory reinnervation of the anesthetic cornea concur with the clinical reports of successful postoperative outcomes in patients with neuropathic keratopathy [3, 5, 10, 11].

The data of this anatomical study described the supratrochlear and supraorbital nerve branches as strong donor nerves, however, the optimal number of donor axons has not yet been determined. The previously reported cut-off point of 900 myelinated axons [14] for donor branch selection in facial reanimation surgery may not be directly translatable to corneal reinnervation surgery, because regeneration distances are significantly longer and the axonal input required for adequate motor reinnervation may differ from the axonal load necessary to restore corneal sensation. The high axon counts of all available trigeminal donor nerves, confirm that distal donor nerve branch selection, which reduced the donor nerve deficit, is possible. The supraorbital nerve proximal to the supraorbital notch contains significantly more myelinated donor axons than its distal branch and the supratrochlear nerve. It remains to be determined if choosing a stronger donor nerve branch for end-to-side coaptation to the nerve graft leads to superior results compared to harvesting a smaller, more distal donor nerve branch for end-to-end coaptation and creating a partial sensory donor nerve deficit.

The different techniques of corneal neurotization involved either direct nerve transfers through a bicoronary dissection [3], or a minimally-invasive technique using small tarsal and supraciliary incisions with interposition of nerve grafts [5, 9]. Recently, Leyngold and colleagues described an endoscopic technique to dissect the supraorbital nerve for corneal neurotization [18, 19]. The nerve fascicles are coapted around the limbus using microsurgical technique in all surgical methods, using either direct nerve transfers or nerve grafting [3, 5, 9]. When autologous nerve grafts were used, the proximal coaptation of the nerve graft to the donor nerve was performed with micro-sutures and fibrin glue [9]. Here, the donor nerve branch selection and coaptation method influences the amount of donor nerve fibers that will grow through the nerve graft and subsequently reinnervate the cornea. As indicated by Elbaz and colleagues, depending on the size of the selected donor nerve branch and the clinical presentation, end-to-side or end-to-end coaptations of the nerve graft can be performed [5]. Larger donor nerve branches are more suitable for end-to-side coaptations, where the end of the nerve graft is sutured to the side of the donor nerve, which preserves donor nerve function [20]. In neuropathic keratopathy, future experimental studies are needed to determine the number of donor nerve axons growing into the nerve graft and reinnervating the cornea. Previous studies have shown that the number of axons growing into nerve grafts is generally less predictable after end-to-side coaptations than after end-to-end coaptations [20, 21]. There are numerous clinical and experimental examples of successful regeneration through end-to-side nerve coaptations [21–28], but in cases of corneal neurotization their applications and benefits compared to end-to-end coaptations remain to be determined. Choosing smaller donor nerve branches and opting for end-to-side coaptations while preserving the continuity of the donor nerve reduces the sensory donor nerve deficit and may be beneficial for the patient, given recent clinical reports of successful corneal neurotization using these less invasive techniques with interposition of nerve grafts [5, 9, 11] compared to the more invasive direct nerve transfer through a bicoronary approach [3]. The additional sensory deficit of the sural nerve graft is limited to the dorsolateral foot and clinically acceptable for the majority of patients [15]. Through improved minimally invasive techniques, the skin incisions in both the donor nerve dissection in the face and the dorsal to the lateral malleolus are limited and the scars are cosmetically acceptable [5]. Benkhatar and colleagues recently proposed using the great auricular nerve instead of the sural nerve graft [12]. The smaller diameter of the nerve graft, which is more compatible to the donor nerve branches than the sural nerve, and the harvest site were cited as main benefits of this technique [12]. The length of the harvested great auricular nerve was 7 cm [12], which is shorter than the sural nerve graft of approximately 15 cm used by other authors [5]. Clinically, the appropriate nerve graft selection will depend on donor nerve selection and anatomical features of the patient, which determine the regeneration distance that needs to be covered.

In the present study, distances to anatomical landmarks for simple donor nerve branch dissection were described. The supratrochlear nerve can be found 2 cm lateral to the facial midline and 2 mm caudal to the orbital rim, whereas the supraorbital donor nerve branch distal to the supraorbital notch can be dissected 1 cm medial to the mid-pupillary line. The described anatomical landmarks are easily clinically applicable and facilitate reliable donor nerve dissection.

Clinical studies demonstrated successful corneal neurotization which improved patients’ vision by avoiding corneal injuries associated with corneal anesthesia and providing improved conditions for subsequent corneal transplantation [5]. After corneal neurotization, in vivo confocal microscopy confirmed the successful reinnervation of stromal and subbasal corneal layers and documented the presence of new sensory nerve fibers [10]. Ting and colleagues underlined the importance of neurotrophic factors providing trophic support after corneal neurotization [11]. It is yet unclear how many ingrowing nerve fibers are required to achieve clinically adequate results. Soluble factors released by trigeminal neurons, which stimulate corneal epithelial cells, and neurotrophic factors expressed by corneal epithelial cells that promote nerve regeneration have been shown to play a crucial role in the pathogenesis of neurotrophic keratopathy [4]. Their role in corneal reinnervation will be the focus of future investigations. The cornea is the most densely innervated surface tissue due to the complex innervation and branching patterns of corneal nerves [29]. Corneal nerves are heterogeneous and innervate different nociceptors: about 20% are mechano-nociceptors, 70% are polymodal nociceptors and 10% are cold-receptors [30]. The role of nociceptor excitation and sensitization after corneal reinnervation remains to be explored in experimental studies and will improve our understanding of the underlying cellular mechanisms of corneal neurotization.

The present study showed that both the supraorbital and the supratrochlear nerve are potent donor nerves that can be reliably dissected using clinical landmarks. Limitations of the study were the advanced age of the cadavers and the limited information about preexisting neurological conditions available from the donors.

## Conclusion

The supraorbital and supratrochlear branches of the trigeminal nerve are potent donor nerves for corneal neurotization, that can be reliably dissected using anatomical landmarks. Restoring sensory innervation to the cornea provides a therapeutic treatment option for patients suffering from neuropathic keratopathy.

## Supporting information

S1 Table Supporting information for nerve biopsies. Histomorphometric analysis of nerve biopsy characterizing fascicle numbers and myelinated axon counts.
(XLSX)

## References

[1] Bonini S, Rama P, Olzi D, Lambiase A. Neurotrophic keratitis. Eye (Lond). 2003;17(8):989–95. 10.1038/sj.eye.6700616 . 14631406

[2] Ramaesh K, Stokes J, Henry E, Dutton GN, Dhillon B. Congenital corneal anesthesia. Surv Ophthalmol. 2007;52(1):50–60. 10.1016/j.survophthal.2006.10.004 . 17212990

[3] Terzis JK, Dryer MM, Bodner BI. Corneal neurotization: a novel solution to neurotrophic keratopathy. Plast Reconstr Surg. 2009;123(1):112–20. 10.1097/PRS.0b013e3181904d3a . 19116544

[4] Muller LJ, Marfurt CF, Kruse F, Tervo TM. Corneal nerves: structure, contents and function. Exp Eye Res. 2003;76(5):521–42. Epub 2003/04/17. . 12697417 10.1016/s0014-4835(03)00050-2

[5] Elbaz U, Bains R, Zuker RM, Borschel GH, Ali A. Restoration of corneal sensation with regional nerve transfers and nerve grafts: a new approach to a difficult problem. JAMA Ophthalmol. 2014;132(11):1289–95. 10.1001/jamaophthalmol.2014.2316 . 25010775

[6] Rosenberg ML. Congenital trigeminal anaesthesia. A review and classification. Brain. 1984;107 (Pt 4):1073–82. . 6509308 10.1093/brain/107.4.1073

[7] Cavanagh HD, Colley AM. The molecular basis of neurotrophic keratitis. Acta Ophthalmol Suppl. 1989;192:115–34. . 2554641 10.1111/j.1755-3768.1989.tb07103.x

[8] Sacchetti M, Lambiase A. Diagnosis and management of neurotrophic keratitis. Clin Ophthalmol. 2014;8:571–9. Epub 2014/03/29. 10.2147/OPTH.S45921 . 24672223 PMC3964170

[9] Bains RD, Elbaz U, Zuker RM, Ali A, Borschel GH. Corneal neurotization from the supratrochlear nerve with sural nerve grafts: a minimally invasive approach. Plast Reconstr Surg. 2015;135(2):397e–400e. 10.1097/PRS.0000000000000994 . 25626824

[10] Fung SSM, Catapano J, Elbaz U, Zuker RM, Borschel GH, Ali A. In Vivo Confocal Microscopy Reveals Corneal Reinnervation After Treatment of Neurotrophic Keratopathy With Corneal Neurotization. Cornea. 2018;37(1):109–12. Epub 2017/10/21. 10.1097/ICO.0000000000001315 . 29053558

[11] Ting DSJ, Figueiredo GS, Henein C, Barnes E, Ahmed O, Mudhar HS, et al Corneal Neurotization for Neurotrophic Keratopathy: Clinical Outcomes and In Vivo Confocal Microscopic and Histopathological Findings. Cornea. 2018 Epub 2018/01/27. 10.1097/ICO.0000000000001522 . 29373338

[12] Benkhatar H, Levy O, Goemaere I, Borderie V, Laroche L, Bouheraoua N. Corneal Neurotization With a Great Auricular Nerve Graft: Effective Reinnervation Demonstrated by In Vivo Confocal Microscopy. Cornea. 2018;37(5):647–50. Epub 2018/02/24. 10.1097/ICO.0000000000001549 . 29474300

[13] Jacinto F, Espana E, Padilla M, Ahmad A, Leyngold I. Ipsilateral supraorbital nerve transfer in a case of recalcitrant neurotrophic keratopathy with an intact ipsilateral frontal nerve: A novel surgical technique. Am J Ophthalmol Case Rep. 2016;4:14–7. Epub 2016/07/18. 10.1016/j.ajoc.2016.07.001 . 29503915 PMC5757463

[14] Terzis JK, Wang W, Zhao Y. Effect of axonal load on the functional and aesthetic outcomes of the cross-facial nerve graft procedure for facial reanimation. Plast Reconstr Surg. 2009;124(5):1499–512. 10.1097/PRS.0b013e3181babb93 . 20009836

[15] Riedl O, Frey M. Anatomy of the sural nerve: cadaver study and literature review. Plast Reconstr Surg. 2013;131(4):802–10. 10.1097/PRS.0b013e3182818cd4 . 23542252

[16] Giovanoli P, Koller R, Meuli-Simmen C, Rab M, Haslik W, Mittlbock M, et al Functional and morphometric evaluation of end-to-side neurorrhaphy for muscle reinnervation. Plast Reconstr Surg. 2000;106(2):383–92. . 10946937 10.1097/00006534-200008000-00021

[17] Tzou CH, Aszmann OC, Frey M. Bridging peripheral nerve defects using a single-fascicle nerve graft. Plast Reconstr Surg. 2011;128(4):861–9. 10.1097/PRS.0b013e31821b6369 . 21921762

[18] Leyngold I, Weller C, Leyngold M, Espana E, Black KD, Hall KL, et al Endoscopic Corneal Neurotization: Cadaver Feasibility Study. Ophthal Plast Reconstr Surg. 2017 Epub 2017/05/05. 10.1097/IOP.0000000000000913 . 28472009

[19] Leyngold I, Weller C, Leyngold M, Tabor M. Endoscopic Corneal Neurotization: Technique and Initial Experience. Ophthal Plast Reconstr Surg. 2018;34(1):82–5. Epub 2017/12/02. . 29194285 10.1097/IOP.0000000000001023

[20] Pannucci C, Myckatyn TM, Mackinnon SE, Hayashi A. End-to-side nerve repair: review of the literature. Restorative neurology and neuroscience. 2007;25(1):45–63. Epub 2007/05/03. . 17473395

[21] Hayashi A, Pannucci C, Moradzadeh A, Kawamura D, Magill C, Hunter DA, et al Axotomy or compression is required for axonal sprouting following end-to-side neurorrhaphy. Exp Neurol. 2008;211(2):539–50. Epub 2008/04/25. 10.1016/j.expneurol.2008.02.031 . 18433746 PMC2761726

[22] Dvali LT, Myckatyn TM. End-to-side nerve repair: review of the literature and clinical indications. Hand Clin. 2008;24(4):455–60, vii Epub 2008/10/22. 10.1016/j.hcl.2008.04.006 . 18928893

[23] Ray WZ, Mackinnon SE. Management of nerve gaps: autografts, allografts, nerve transfers, and end-to-side neurorrhaphy. Exp Neurol. 2010;223(1):77–85. Epub 2009/04/08. 10.1016/j.expneurol.2009.03.031 . 19348799 PMC2849924

[24] Tarasidis G, Watanabe O, Mackinnon SE, Strasberg SR, Haughey BH, Hunter DA. End-to-side neurorrhaphy resulting in limited sensory axonal regeneration in a rat model. Ann Otol Rhinol Laryngol. 1997;106(6):506–12. 10.1177/000348949710600612 . 9199612

[25] Placheta E, Wood MD, Lafontaine C, Liu EH, Hendry JM, Angelov DN, et al Enhancement of facial nerve motoneuron regeneration through cross-face nerve grafts by adding end-to-side sensory axons. Plast Reconstr Surg. 2015;135(2):460–71. 10.1097/PRS.0000000000000893 . 25626793

[26] Frey M, Giovanoli P, Michaelidou M. Functional upgrading of partially recovered facial palsy by cross-face nerve grafting with distal end-to-side neurorrhaphy. Plast Reconstr Surg. 2006;117(2):597–608. 10.1097/01.prs.0000197136.56749.c6 . 16462346

[27] Viterbo F, Franciosi LF, Palhares A. Nerve graftings and end-to-side neurorrhaphies connecting the phrenic nerve to the brachial plexus. Plast Reconstr Surg. 1995;96(2):494–5. . 7624435 10.1097/00006534-199508000-00054

[28] Viterbo F, Sanches J, T RW. Clinical applications of the end-to-side neurorrhaphy. In: Frey M, Giovanoli P, Koller R, editors. 5^th^ International Muscle Symposium. 1st ed. Vienna2000. p. 151–4.

[29] Marfurt CF, Cox J, Deek S, Dvorscak L. Anatomy of the human corneal innervation. Exp Eye Res. 2010;90(4):478–92. Epub 2009/12/29. 10.1016/j.exer.2009.12.010 . 20036654

[30] Belmonte C, Acosta MC, Gallar J. Neural basis of sensation in intact and injured corneas. Exp Eye Res. 2004;78(3):513–25. Epub 2004/04/27. . 15106930 10.1016/j.exer.2003.09.023

