# Correction: Axon numbers and landmarks of trigeminal donor nerves for corneal neurotization

**DOI:** 10.1371/journal.pone.0211748

**Published:** 2019-02-28

**Authors:** The PLOS ONE Staff

The name of the second author appears incorrectly in the citation. The correct citation is: Györi E, Tzou CH, Weninger WJ, Reissig L, Schmidt-Erfurth U, Radtke C, et al. (2018) Axon numbers and landmarks of trigeminal donor nerves for corneal neurotization. PLoS ONE 13(10): e0206642. https://doi.org/10.1371/journal.pone.0206642
